# Factor XIII Deficiency: A Rare Cause of Hydrocephalus in Infancy

**DOI:** 10.1002/ccr3.70962

**Published:** 2025-10-07

**Authors:** Ahmed Alali, Thkra Meshal, Zahraa Alali, Sarah Hamed, Manar Ali, Kareem lbraheem

**Affiliations:** ^1^ Faculty of Medicine Mansoura University Mansura Egypt; ^2^ Faculty of Medicine Palestine Polytechnic University Hebron Palestine; ^3^ College of Medicine and Medical Sciences Arabian Gulf University Manama Bahrain

**Keywords:** factor XIII deficiency, hydrocephalus, intracranial hemorrhage (ICH), neonatal bleeding disorders

## Abstract

Factor XIII deficiency is a very rare bleeding disorder that can cause serious bleeding problems, especially in infants. We describe a 5‐month‐old girl who came in with a rapidly increasing head size and a bulging fontanelle. She had no fever or injury. A CT scan showed bleeding in the right side of her brain and hydrocephalus. Her factor XIII level was low, about 25%, while other clotting factors were normal. Her older brother had the same condition and unfortunately died at 9 months due to similar brain bleeding and infection. She was treated with plasma products to replace the missing factor, antibiotics, and seizure medications. After treatment, her condition improved and she was discharged with plans for close follow‐up. This case shows how factor XIII deficiency can lead to life‐threatening bleeding and hydrocephalus. Early diagnosis and treatment are crucial, and knowing the family history was important here. She will need ongoing monitoring to catch any future bleeding or complications early.

## Graphical abstract

Xx.

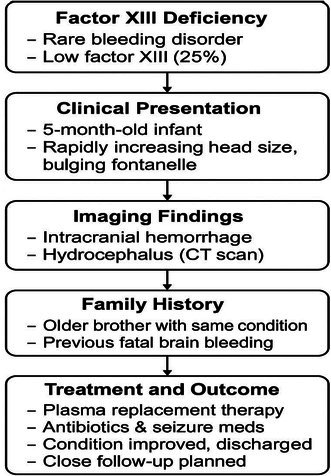

**Summary**

- Factor XIII (FXIII) deficiency can lead to fetal complications such as intracranial hemorrhage therefore, early diagnosis and treatment are crucial.
- A family history of factor XIII deficiency even without consanguinity should raise suspicion.
- A 5‐month old female patient presented with hydrocephalus due to intracranial hemorrhage with head circumference of 52 cm and her bleeding tendency screening revealed 25% decrease of factor XIII.
- The patient was treated with antibiotics, antiseizure medication and thrombex gel.

## 1 Introduction

Factor XIII (FXIII) deficiency is a rare inherited bleeding disorder characterized by deficiency of clotting factor XIII resulting in varying bleeding manifestations depending on the plasma level of factor XIII including delayed detachment and bleeding of the umbilical cord which is observed in 80% of cases, easily bruising and soft tissue bleeding, intracranial hemorrhage in 30% of cases [1], hemarthrosis, muscle hematoma, prolonged bleeding after trauma or surgery, heavy menstrual bleeding and recurrent early pregnancy loss [2].

Factor XIII deficiency occurs in approximately 1 in 2–3 million live births, affecting males and females equally [2]. FXIII is an autosomal recessive disorder due to mutations in *F13A1* gene on the short arm of chromosome 6 and *F13B* gene located on the long arm of chromosome 1, with mutations in *F13B* being less common and associated with milder manifestations. Factor XIII plays an essential role in stabilizing clots and preventing clots breakdown therefore reducing the risk of prolonged and uncontrollable bleeding [3].

Hydrocephalus is a neurological disorder characterized by accumulation of cerebospinal fluid in the brain, it can arise due to numerous causes including bleeding disorders complicated by intraventricular hemorrhage, head trauma, stroke, tumor and meningitis. Patients present with enlarged head, bulging anterior fontanelle, sunsetting eyes, and signs of increased intracranial pressure such as headache, blurring of vision, and projectile vomiting [4].

## 2 Case Presentation

A 5‐month‐old female with a history of factor XIII deficiency was evaluated. On physical examination, she exhibited a tense and bulging anterior fontanelle without associated fever, weight loss, or bleeding tendency. Since birth, she had been admitted to the neonatal intensive care unit (NICU) as an infant of a diabetic mother (IDM) and was noted to have an increasing head circumference. Screening for a bleeding tendency revealed a 25% decrease in factor XIII, while factors VIII, IX, and X were within normal limits, and she was subsequently discharged. Her family history revealed no consanguinity, although her brother, also diagnosed with factor XIII deficiency, had developed similar symptoms, with his head circumference reaching 54 cm. He succumbed to intracranial hemorrhage (ICH), sepsis, and disseminated intravascular coagulation (DIC) at 9 months of age after admission to the pediatric ICU (PICU).

**FIGURE 1:**
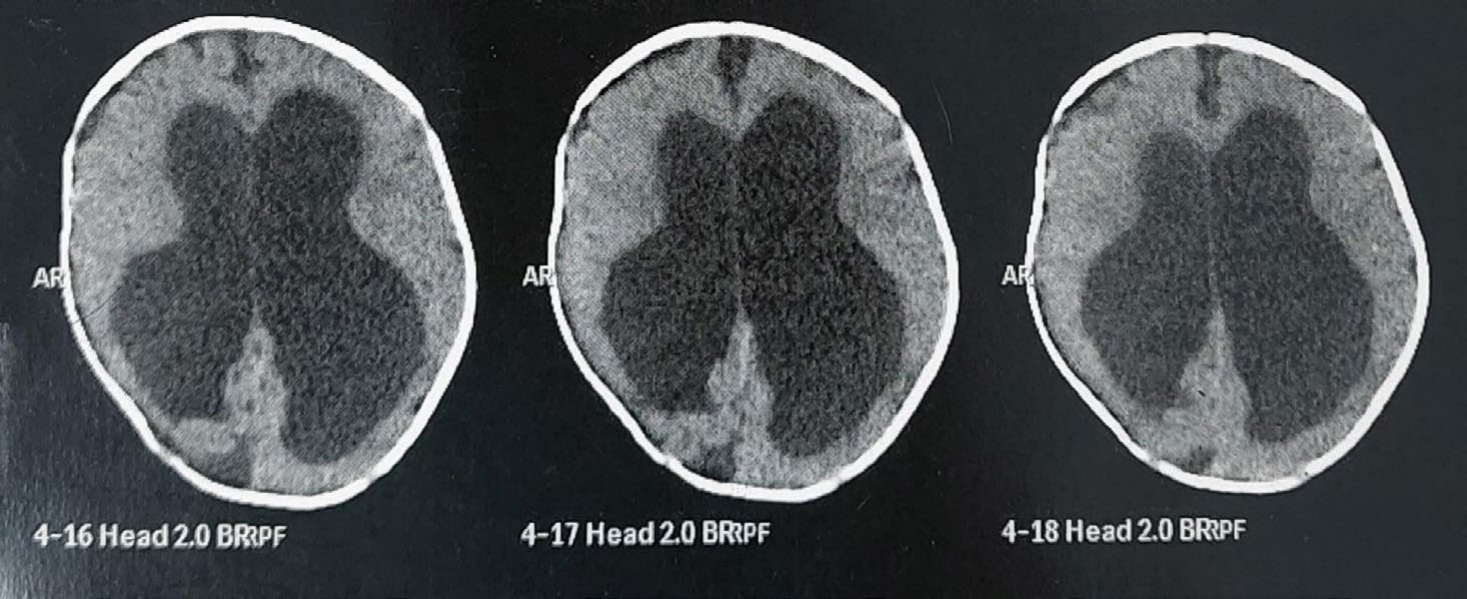
A CT scan showed the presence of extra‐axial hypodense areas on the right side of the brain, exerting a mass effect, compressing the ipsilateral ventricle, and causing a midline shift.

The differential diagnosis for the patient's condition included hydrocephalus secondary to congenital abnormalities, trauma, infection, or hemorrhage due to her factor XIII deficiency. A CT scan showed right‐sided ICH and ventriculomegaly (Figure 1). Initial laboratory investigations revealed normocytic normochromic anemia with a hemoglobin level of 11.4 g/dL, leukocytosis (WBC count of 19.5), and thrombocytosis (platelet count of 631 k/μL) (Table 1). A non‐contrast CT scan demonstrated marked supra‐ and infratentorial hydrocephalus with significant brain atrophy, thinning of the white matter, and evidence of mild intraventricular hemorrhage. The cerebrospinal fluid (CSF) was examined repeatedly, and cytological analysis was negative for malignancy (Table 2). Additional laboratory results showed a serum calcium level of 8.8 mg/dL, negative C‐reactive protein (CRP), and an elevated erythrocyte sedimentation rate (ESR) of 40 mm/h.

**TABLE 1:** Complete blood count values.

Complete blood count	Reference range	Day 1	Day 5	Day 11	Day 11	Day 13
Hemoglobin (g/dL)	12–18	11.4	9.75	10.8	11.4	11.3
HCT	36%–48%	32.2%	26.9%	29.3%	32.4%	37.2
Total leucocyte count (k/μL)	4.4–12.9	19.5	11.6	15.1	19.1	14.3
MCV (fL)	80–97	81.7	80	78	81.3	81.2
MCH (pg)	26–32	29	29	28	28.7	27.9
Platelet count (k/μL)	140–440	6331	506	404	235	360

**TABLE 2:** CSF analysis.

Body fluid (CSF)	Reference range	Day 2	Day 4	Day 6
Physical properties				
Color	Colorless	Reddish	Dark yellowish	Yellowish
Aspect	Clear	Turbid	S.turbid	S.turbid
pH			7.4	7.5
Microscopic examination				
Total protein (mg/dL)	20–40		39	46
Glucose (mg/dL)	60–100	51	70	74
LDH (U/L)	0–100		96	139
CL (mmol/L)	110–130		119	114
WBCs	0–5	50	4	4
Granulocyte (%)			60	65
Lymphocyte (%)			40	35

## 3 Results/Follow‐Up

The patient subsequently developed hydrocephalus, marked by a head circumference of 52 cells/mm^3^, secondary to ICH. She received one unit of fresh frozen plasma (FFP) and two units of cryoprecipitate. Empirical therapy included intravenous antibiotics—Unasyn (ampicillin/sulbactam) 700 mg every 8 h and Claforan (cefotaxime) 1 g every 12 h—as well as antiepileptic medication, Levetiracetam (500 mg every 12 h). Additional supportive care included topical thrombin (Thrombex Gel) and zinc oxide.

After 12 days of hospitalization, the patient showed clinical improvement and was discharged on a regimen of Levetiracetam, Meropenem (500 mg), Depakine drops (valproic acid), Thrombex Gel, and zinc oxide, all to be administered every 12 h. Continued outpatient follow‐up was advised due to the underlying coagulopathy and high risk of recurrence. Her prognosis remains guarded given the family history of fatal outcomes in her sibling with the same condition.

## 4 Discussion

The case of a 5‐month‐old female with factor XIII deficiency presenting to hospital with hydrocephalus due to intracranial hemorrhage (ICH) highlights the severe implications of this rare bleeding disorder. Factor XIII deficiency is known to predispose individuals to significant bleeding complications like prolonged umbilical cord bleeding, mucosal bleeding and ICH, which can lead to bad outcomes, including neurological damage and mortality [4, 5]. In this case, the patient's head circumference of 52 cm and the presence of a tense, bulging anterior fontanelle are indicative of increased intracranial pressure, which is a common consequence of ICH and hydrocephalus [6]. The diagnostic imaging was done and the CT scan revealed hypodense areas compressing the ipsilateral ventricle, causing a midline shift. This finding is compatible with previous reports that describe similar patterns of ICH in patients with factor XIII deficiency, where the hemorrhage often occurs in the supratentorial region, resulting in significant mass effect and subsequent hydrocephalus [5, 6].

The family history of a sibling with a similar condition who died due to ICH highlights the hereditary nature of factor XIII deficiency and its potential for severe clinical manifestations [7]. Laboratory findings in this case revealed a 25% decrease in factor XIII levels, which aligns with the known pathophysiology of the disorder. Factor XIII plays a crucial role in stabilizing fibrin clots, and its deficiency can lead to inadequate hemostasis, predisposing patients to bleeding events, including ICH [4, 7]. Her laboratory tests showing normocytic normochromic anemia and leukocytosis further suggest a complex clinical picture, possibly indicating an underlying inflammatory or infectious process, which is common in cases of severe hemorrhage [6].

Management of this patient involved a multidisciplinary approach, including the administration of antibiotics and antiepileptic medications, reflecting the need to address both the infectious risk and the neurological complications associated with ICH [4, 5]. The patient received 1 unit of fresh frozen plasma and 2 units of cryoprecipitate, which are essential for managing factor XIII deficiency [7]. The use of supportive therapies, such as zinc olive ointment and fibrin sealant, indicates an effort to manage the bleeding tendency and support overall recovery [7]. The patient's discharge after 12 days on a regimen that included antiepileptics and antibiotics suggests a stabilization of her condition, arteriovenous shunt was performed, although long‐term follow‐up will be essential to monitor for potential sequelae of her ICH and hydrocephalus [4, 5].

This case is consistent with findings from an Iranian cohort study, which reported severe bleeding episodes, including ICH, in patients with factor XIII deficiency [8], A study by Alavi et al. [4] emphasized the high risk of spontaneous ICH in children with bleeding disorders, underscoring the need for early diagnosis and intervention.

## Author Contributions

**Ahmed Alali:** conceptualization, data curation, investigation, methodology, writing – original draft, writing – review and editing. **Thkra Meshal:** data curation, investigation, software, writing – original draft, writing – review and editing. **Zahraa Alali:** data curation, formal analysis, project administration, writing – original draft. **Sarah Hamed:** conceptualization, formal analysis, writing – review and editing. **Manar Ali:** data curation, investigation, writing – review and editing. **Kareem lbraheem:** conceptualization, data curation, supervision, validation, writing – original draft, writing – review and editing.

## Consent

Written informed consent was obtained from the patient parents for the publication of this case report.

## Conflicts of Interest

The authors declare no conflicts of interest.

## Data Availability

The data used to support the findings of this study are included in the article.
